# The NLRP1 Emerges as a Promising Therapeutic Target and Prognostic Biomarker Across Multiple Cancer Types: A Comprehensive Pan‐Cancer Analysis

**DOI:** 10.1002/cam4.70836

**Published:** 2025-04-16

**Authors:** Leila Habibipour, Mahboubeh Sadeghi, Alireza Raghibi, Nima Sanadgol, Amirhossein Mohajeri Khorasani, Pegah Mousavi

**Affiliations:** ^1^ Molecular Medicine Research Center, Hormozgan Health Institute Hormozgan University of Medical Sciences Bandar Abbas Iran; ^2^ Department of Medical Genetics, Faculty of Medicine Hormozgan University of Medical Sciences Bandar Abbas Iran; ^3^ Student Research Committee Hormozgan University of Medical Sciences Bandar Abbas Iran; ^4^ Department of Medical Genetics, School of Medicine Tehran University of Medical Sciences Tehran Iran; ^5^ Institute of Neuroanatomy RWTH University Hospital Aachen Aachen Germany; ^6^ Medical Genetics Research Center Mashhad University of Medical Sciences Mashhad Iran; ^7^ Metabolic Syndrome Research Center Mashhad University of Medical Sciences Mashhad Iran; ^8^ Student Research Committee Mashhad University of Medical Sciences Mashhad Iran

**Keywords:** biomarker, NLRP1, pan‐cancer, prognosis, treatment

## Abstract

**Introduction:**

Nod‐like receptor family pyrin domain containing 1 (NLRP1) serves as the central component of the inflammasome complex and has emerged as a potential contributor to cancer development. Despite accumulating evidence, a comprehensive assessment of NLRP1 across various cancer types has yet to be undertaken.

**Methods:**

Several databases have evaluated NLRP1 expression across various cancer types in The Cancer Genome Atlas (TCGA). Additionally, studies have investigated the correlation between NLRP1 and various survival metrics, infiltration of cancer‐associated fibroblasts, genetic alterations, drug sensitivity, and promoter methylation. Furthermore, research has explored the potential roles of NLRP1 and its interactions with other proteins.

**Results:**

Our analysis revealed decreased expression of NLRP1 in BLCA, BRCA, KICH, LUAD, LUSC, PRAD, and UCEC tumor tissues compared to normal tissues. We identified a significant correlation between NLRP1 expression and various cancer survival parameters, genetic mutations, and immune infiltration of cancer‐associated fibroblasts. Furthermore, we observed that NLRP1 expression is regulated by promoter DNA methylation in ESCA. Abnormal expression of NLRP1 was associated with decreased sensitivity to multiple anti‐tumor drugs and small compounds. NLRP1 was found to be involved in pathways associated with T cell receptors and chemokines.

**Conclusions:**

Reduced NLRP1 expression contributes to cancer progression and holds potential as a crucial biomolecular marker for diagnostic, prognostic, and personalized therapeutic interventions across different malignancies.

## Graphical abstract

NLRP1, a key component of the inflammasome complex, has emerged as a significant factor in cancer development. Through a systematic in silico study, we observed NLRP1 dysregulation in multiple tumor types, with significant correlations to survival outcomes, cancer‐associated fibroblast infiltration, and drug sensitivity, highlighting its potential as a biomarker for cancer diagnosis, prognosis, and personalized treatment strategies.

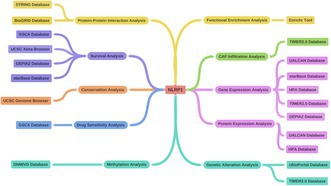

**Abbreviations**
ACC: adrenocortical carcinoma
BLCA: bladder urothelial carcinoma
BRCA: breast invasive carcinoma
CAF: cancer‐associated fibroblast
CESC: cervical squamous cell carcinoma and endocervical adenocarcinoma
CHOL: Cholangio Carcinoma
COAD: Colon Adenocarcinoma
CPTAC: Clinical Proteomic Tumor Analysis Consortium
CTRP: Cancer Therapeutics Response Portal
DAMP: danger‐associated molecular patterns
DFS: disease‐free survival
DLBC: lymphoid neoplasm diffuse large B‐cell lymphoma
DSS: disease‐specific survival
ENCORI: Encyclopedia of RNA Interactomes
ESCA: Esophageal carcinoma
FANTOME5: function annotation of the mammalian genome
FDR: false discovery rate
FKLC: familial keratosis lichenoides chronica
GBM: glioblastoma multiforme
GEPIA2: Gene Expression Profiling Interactive Analysis version 2
GO: Gene Ontology
GSCA: Gene Set Cancer Analysis
GTEx: Genotype‐Tissue Expression
HNSC: head and neck squamous cell carcinoma
HPA: The Human Protein Atlas
IL: interleukin
KEGG: Kyoto Encyclopedia of Genes and Genomes
KICH: Kidney Chromophobe
KIRC: Kidney Renal Clear Cell Carcinoma
KIRP: Kidney Renal Papillary Cell Carcinoma
LAML: acute myeloid leukemia
LGG: brain lower grade glioma
LIHC: liver hepatocellular carcinoma
LUAD: lung adenocarcinoma
LUSC: lung squamous cell carcinoma
MESO: mesothelioma
MSPC: multiple self‐healing palmoplantar carcinoma
NF‐κB: nuclear factor‐κB
NK: natural killer
NLRP1: NOD‐like receptor family pyrin domain containing 1
OS: overall survival
OV: ovarian serous cystadenocarcinoma
PAAD: pancreatic adenocarcinoma
PAMP: pathogen‐associated molecular patterns
PCPG: pheochromocytoma and paraganglioma
PFS: progression‐free survival
PPI: protein–protein interaction
PRAD: prostate adenocarcinoma
qRT‐PCR: quantitative real‐time polymerase chain reaction
READ: rectum adenocarcinoma
SARC: sarcoma
SKCM: skin cutaneous melanoma
STAD: stomach adenocarcinoma
TCGA: The Cancer Genome Atlas
TGCT: testicular germ cell tumors
THCA: thyroid carcinoma
THYM: thymoma
TIMER2.0: Tumor Immune Estimation Resource version 2
UALCAN: University of Alabama at Birmingham CANcer data analysis Portal
UCEC: Uterine Corpus Endometrial Carcinoma
UCS: uterine carcinosarcoma
UVM: uveal melanoma

## 1 Introduction

Cancer is a disease characterized by uncontrolled cell growth, often resulting in significant mortality rates worldwide [1]. Examining various facets of carcinogenesis could aid in the discovery of potential new therapeutic agents. High‐throughput experimental methods have generated multi‐omics cancer datasets, allowing for the analysis of extensive genomic data [2]. The Cancer Genome Atlas (TCGA) stands as one of the paramount multi‐omics cancer‐associated databases, offering a wealth of molecular aberration information across a wide array of malignancies. Additionally, user‐friendly TCGA‐derived databases are readily available, empowering researchers to conduct pan‐cancer analyses [3].

Nod‐like receptor family pyrin domain containing 1 (NLRP1) is situated on human chromosome 17p13.2 and encodes a 155 kilodalton (kDa) protein [4]. As a fundamental component of the cytosolic inflammasome, NLRP1 forms multiprotein oligomers within the innate immune system. In mammals, signals from pathogen‐associated molecular patterns (PAMPs) and danger‐associated molecular patterns (DAMPs) induce the oligomerization of NLRP1 and the assembly of inflammasomes. Inflammasomes are accountable for the inflammatory form of cell death known as pyroptosis [5]. Furthermore, the assembly and activation of the inflammasome lead to the activation of caspase‐1, which, in turn, triggers the proteolytic cleavage, maturation, and secretion of proinflammatory cytokines, including interleukin 18 (IL‐18) and interleukin 1β (IL‐1β) [6]. Polymorphisms in the inflammasome sensor NLRP1 have been linked to cancer susceptibility, including mesothelioma [7], melanoma [8], and epidermal hyperplasia [9]. Additionally, germline mutations of NLRP1 are associated with two human skin disorders: familial keratosis lichenoides chronica (FKLC) and multiple self‐healing palmoplantar carcinoma (MSPC). Zhong et al. proposed that these mutations may enhance the self‐oligomerization of NLRP1, thereby increasing its activation. Moreover, besides inflammasome activation, IL‐1 signaling promotes epidermal hyperplasia and skin inflammation, thereby heightening susceptibility to skin cancer [9]. A prior investigation in breast cancer revealed a significant positive correlation between the transcriptional level of NLRP1 and Ki‐67 levels, TNM stage, and lymph node metastasis. The study demonstrated that NLRP1 overexpression augmented breast cancer cell proliferation, migration, and invasion [10]. Another study suggests that the anticancer effects of NLRP1 are associated with its reduced mRNA and protein expression, possibly as a result of methylation, in cutaneous squamous cell carcinomas [11]. Hence, there is ongoing debate regarding the role of NLRP1 in tumorigenesis.

Considering the significant role of NLRP1 in cancer pathogenesis and the existing uncertainty surrounding its alterations in various cancers, this study was undertaken. The paper investigates the expression pattern of NLRP1 and its associated survival prognosis across multiple tumor types using data from TCGA and several cancer‐associated databases. Furthermore, correlations between NLRP1 expression and immune infiltration were explored. The TCGA cancer types and Genotype‐Tissue Expression (GTEx) normal tissues included in the study are thoroughly detailed in Data S1.

## 2 Materials and Methods

### 2.1 Gene Expression Analysis in Normal Tissues

The Human Protein Atlas (HPA) (https://www.proteinatlas.org/) contains protein and mRNA expression profiles of genes in organs, tissues, and cells using various omics technologies, such as mass spectrometry‐based proteomics, antibody‐based imaging, and transcriptomics. It provides great pan‐cancer information through tissue and pathology modules. We made an NLRP1 mRNA expression plot in normal tissues utilizing the HPA database (version: 23.0) [12].

### 2.2 Gene Expression Analysis in Cancerous Tissues Compared to Normal Tissues

The transcriptional level of NLRP1 was also searched in cancerous tissues compared to normal tissues through GEPIA2, TIMER2.0, UALCAN, and starBase v2.0 databases. The Gene Expression Profiling Interactive Analysis version 2 (GEPIA2); (http://gepia2.cancer‐pku.cn/#analysis) is a search tool widely used for gene expression analysis of different tumors and normal samples from the TCGA and the GTEx data. This database provides useful information, such as differential expression analysis profile, survival prognosis analysis, correlation analysis, similar gene detection, expression profile boxplot, and stage plot of the desired gene [13]. The Tumor Immune Estimation Resource version 2 (TIMER2.0) (http://timer.cistrome.org/) is an integrative database with unique features of tumor immunity infiltration analysis and differential gene expression analysis [14]. The University of ALabama at Birmingham CANcer data analysis Portal (UALCAN) (http://ualcan.path.uab.edu/analysis.html) is an interactive search tool that enables the user to access valuable cancer OMICS data, such as TCGA, and Clinical Proteomic Tumor Analysis Consortium (CPTAC). Exploring the interest gene, UALCAN provides a pan‐cancer gene expression profile, and survival analysis [15]. starBase v2.0 (http://starbase.sysu.edu.cn/panCancer.php) or the Encyclopedia of RNA Interactomes (ENCORI) is a web service designed for exploring the interaction among different cellular RNA. It also provides a pan‐cancer module to analyze the differential expression and survival of the interest gene [16]. We also applied the “Pathological Stage Plot” section of GEPIA2 to assess the association between NLRP1 transcriptional level and cancer stages in all TCGA tumors.

### 2.3 Protein Expression Analysis

UALCAN was utilized to explore the NLRP1 expression analysis with the source of the CPTAC dataset. We carried out the total protein expression level of the primary tumor in comparison to normal tissue by entering “NLRP1”. The available cancer information for the NLRP1 gene was included from the CPTAC dataset, consisting of breast cancer, Lung adenocarcinoma, Head and neck squamous carcinoma, and Pancreatic adenocarcinoma. The HPA database proved to be a valuable resource in investigating the protein expression patterns in healthy tissues, which was a significant advantage.

### 2.4 Conservation and Survival Analysis

Utilizing the UCSC genome browser (GRCh38/hg38 human genome assembly; https://genome.ucsc.edu/cgi‐bin/hgGateway) [17, 18], we reached the NLRP1 gene conservation among vertebrates. The Gene Set Cancer Analysis (GSCA) platform (http://bioinfo.life.hust.edu.cn/GSCA/#/expression) integrates genomics, pharmacogenomics, and immunogenomic datasets for a comprehensive study of gene sets in cancer [19, 20]. In our investigation, we utilized the GSCA database to assess the impact of NLRP1 expression on overall survival (OS), progression‐free survival (PFS), disease‐free survival (DFS), and disease‐specific survival (DSS) outcomes in patients with various tumors. Additionally, the UCSC Xena Browser (https://xenabrowser.net/) was employed to evaluate the association of NLRP1 with OS, PFS, DFS, and DSS [21]. The GEPIA2 database was also utilized to analyze OS and DFS. The “Survival Map” section of GEPIA2 was implemented to achieve the OS and DFS importance map data associated with NLRP1 among all tumors. Furthermore, the starBase v2.0 database was employed to investigate NLRP1‐related OS in all TCGA cancers. A log‐rank *p*‐value < 0.05 was employed to denote statistical significance across all databases. Finally, we performed an intersection analysis of data from these databases to evaluate the prognostic potential of NLRP1 transcriptional level across different malignancies.

### 2.5 Drug Sensitivity Analysis

To assess the potential clinical relevance of abnormal NLRP1 gene expression and its viability as a predictive biomarker for drug screening, we carried out a drug sensitivity analysis using the GSCA database. Our analysis involved collecting the IC50 values of 481 Chemotherapeutic agents or medicines across various cell lines, along with their RNA transcription profiles from the Cancer Therapeutics Response Portal (CTRP) dataset within the GSCA database. Using this database, Pearson correlation analysis was executed to ascertain the degree of association between RNA expression profile and medicine IC50 to determine the relationship between gene expression and drug sensitivity, considering a False Discovery Rate (FDR) adjustment method to ensure the validity of the results. A negative correlation would suggest that increased expression of the gene is associated with reduced drug sensitivity or potential resistance, and conversely.

### 2.6 Co‐Expression Analysis

The Enrichr online tool (https://maayanlab.cloud/Enrichr/) [22, 23, 24] was deployed to identify the principal 100 genes co‐expressed with NLRP1, utilizing the ARCHS4 RNA‐seq gene–gene co‐expression matrix (Data S2).

### 2.7 Functional Enrichment Analysis

Enrichment analysis was performed on a cohort of the mentioned 100 genes to investigate the potential functional implications of genes co‐expressed with NLRP1 in different cancer types. This analysis included Gene Ontology (GO) enrichment across the domains of cellular components, molecular function, and biological process. Furthermore, pathway enrichment analysis was conducted, incorporating data from the Kyoto Encyclopedia of Genes and Genomes (KEGG), Wikipathway, and Reactome databases. These enrichment processes were facilitated by the Enrichr tool with a defined significance threshold established at a *p*‐value less than 0.05. To graphically display the results of the GO and pathway enrichment analyses, dot plots were generated utilizing the ggplot2 package [25] within the R programming environment (version R‐4.2.1, 64‐bit, https://www.r‐project.org/) [26] and RStudio Desktop (version 2022.7.0.548) [27].

### 2.8 Cancer‐Associated Fibroblast (CAF) Infiltration Analysis

We utilized the TIMER2.0 database to investigate the correlation between NLRP1 expression and the infiltration of CAF across diverse cancer types. Specifically, we employed the “Immune‐Gene” section of TIMER2.0 to assess the association between NLRP1 transcriptional level and CAF infiltration in 32 TCGA neoplasms. To estimate immune infiltration, we utilized the “EPIC” algorithm in conjunction with a partial Spearman's correlation test (purity adjustment). A *p*‐value < 0.05 was considered the statistical significance cutoff.

### 2.9 Genetic Alteration Analysis

The assessment of NLRP1 genetic alterations was conducted using cBioPortal (https://www.cbioportal.org/) [28]. Specifically, within the “Quick Choose” section, we opted for “TCGA Pan‐Cancer Atlas Studies” to scrutinize the genetic alteration characteristics of the NLRP1 gene in all TCGA cancers. Entering “NLRP1” into the “Query” section, we set the molecular profiles to include “mutations” and “copy number alterations”. The “Cancer Types Summary” module provides us with the various NLRP1 genetic alteration types including mutation, amplification, deep deletion, and the existence of multiple alterations in various TCGA cancers. Moreover, we examined the types of NLRP1 gene mutations across various cancers using the “mutations” module. Furthermore, we obtained an overview of cancerous samples with NLRP1 mutations in each tumor type from the “Gene_Mutation” module of the TIMER2.0 database.

### 2.10 Protein–Protein Interaction Analysis

The STRING database version 12 (https://string‐db.org/) is a platform designed for the exploration and analysis of protein–protein interaction (PPI) data, facilitating the identification of direct (physical) and indirect (functional) interactions among proteins [29]. Our objective was to acquire PPI network information for NLRP1 by setting the interaction score cutoff to 0.15 and activating all interaction sources, evidence network edge, and a maximum of 100 interactors as the basic settings. Additionally, we utilized the BioGRID version 4.4.228 (https://thebiogrid.org), a curated biological open‐access database that provides information on protein–protein interactions, chemical, and genetic interactions, as well as post‐translational modifications, to explore validated physical PPI [30]. The ‘Network’ module of this database was employed, with the layout set to ‘Concentric Circles’. Furthermore, we employed the Venny 2.1.0 tool (https://bioinfogp.cnb.csic.es/tools/venny/) to ascertain the overlap between the STRING and BioGRID databases, thereby identifying the NLRP1 PPIs that were mentioned in the literature [31].

### 2.11 Methylation Analysis

DNMIVD (http://119.3.41.228/dnmivd/index/) is an interactive database designed for the exploration of DNA methylation patterns sourced from the Gene Expression Omnibus (GEO) and TCGA databases [32, 33]. The database provides the methylation status of the NLRP1 gene across all available TCGA cohorts. To compare methylation levels between cancer and normal samples, an independent Student's *t*‐test was employed, considering cancers with |beta difference| > 0.2 and an independent Student's *t*‐test adjusted *p*‐value < 0.05 as tumors exhibiting significant changes in the NLRP1 gene promoter. Furthermore, in instances where abnormal methylation of the NLRP1 gene promoter was observed, we investigated the relationship between gene expression and promoter methylation in primary tissues using Pearson and Spearman correlation analyses, applying predefined criteria (|*R* value| > 0.1 and *p*‐value < 0.05).

## 3 Results

### 3.1 Gene Expression Analysis in Normal Tissues

**FIGURE 1:**
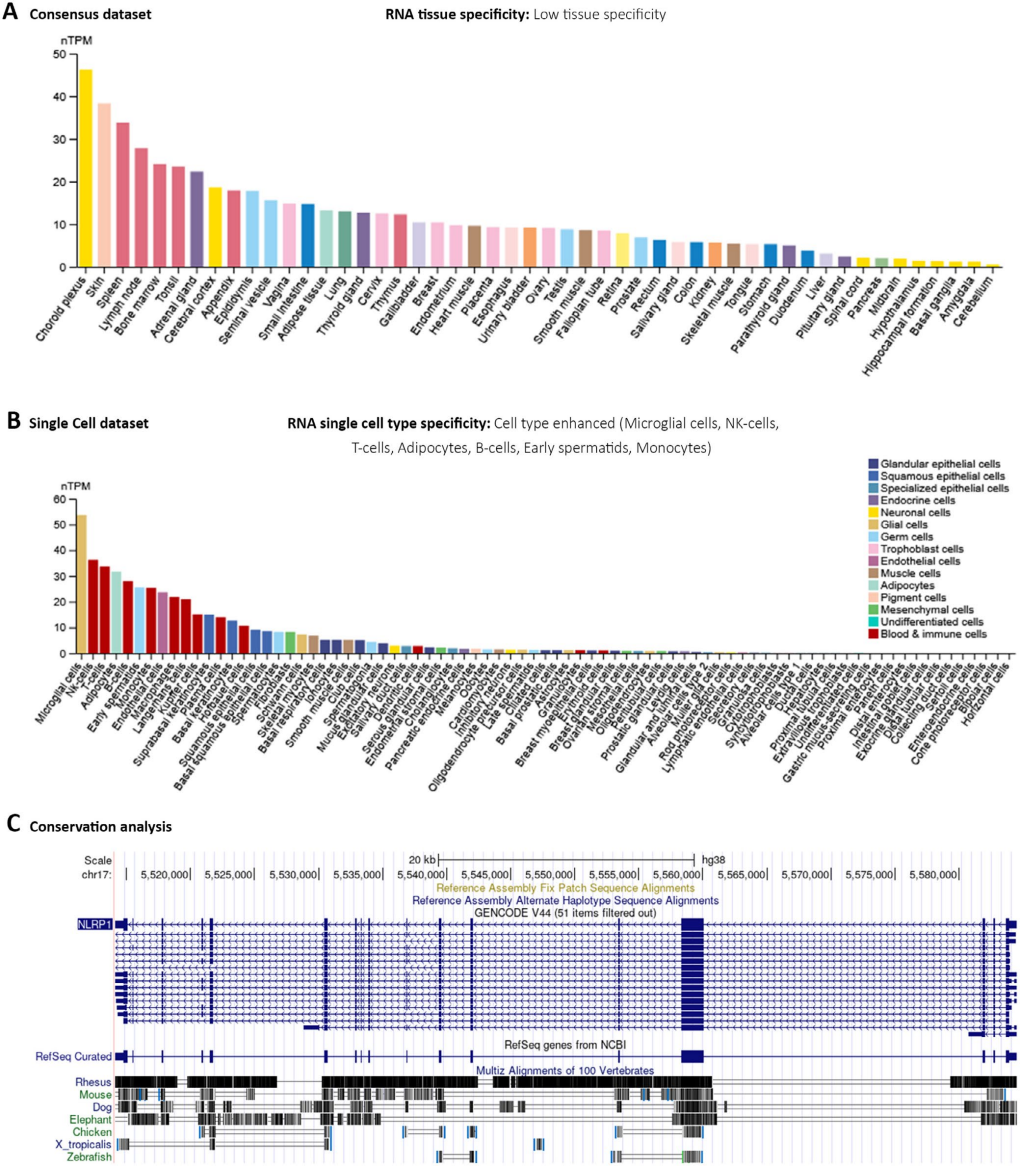
The intricate NLRP1 expression pattern across diverse normal tissues and cells along with an exploration of NLRP1 gene conservation among vertebrates. (A) The Consensus NLRP1 tissue expression is based on datasets of HPA, and GTEx. (B) The expression status of NLRP1 in single‐cell types by HPA. (C) The gene conservation analysis of NLRP1 among vertebrates.

The function annotation of the mammalian genome (FANTOME5), in tandem with the HPA, GTEx project, and a hybridized Consensus dataset, which combines the insights from HPA and GTEx, has been scrutinized within the HPA database framework. This critical examination revealed an elevated expression of the NLRP1 gene in diverse anatomical structures, such as the Choroid plexus, skin, spleen, lymphoid nodes, bone marrow, tonsil, and appendix (Figure 1A and Figure S1). These observations suggest a low degree of tissue specificity of the NLRP1, quantitatively supported by a Tau specificity score of 0.38.

At the granularity of single‐cell analyses, NLRP1 manifests a high‐expression profile within a range of cell types, encompassing microglia in the central nervous system, natural killer (NK) cells, adaptive immune T‐cells, adipose cells, B‐cell lineages of the immune system, early spermatids, and monocytic cells (Figure 1B). Parallel genomic assessment using the UCSC Genome Browser has demonstrated a modest level of evolutionary conservation of the NLRP1 gene across the vertebrate taxa (Figure 1C).

### 3.2 Gene Expression Analysis in Cancerous Tissues Compared to Normal Tissues

**FIGURE 2:**
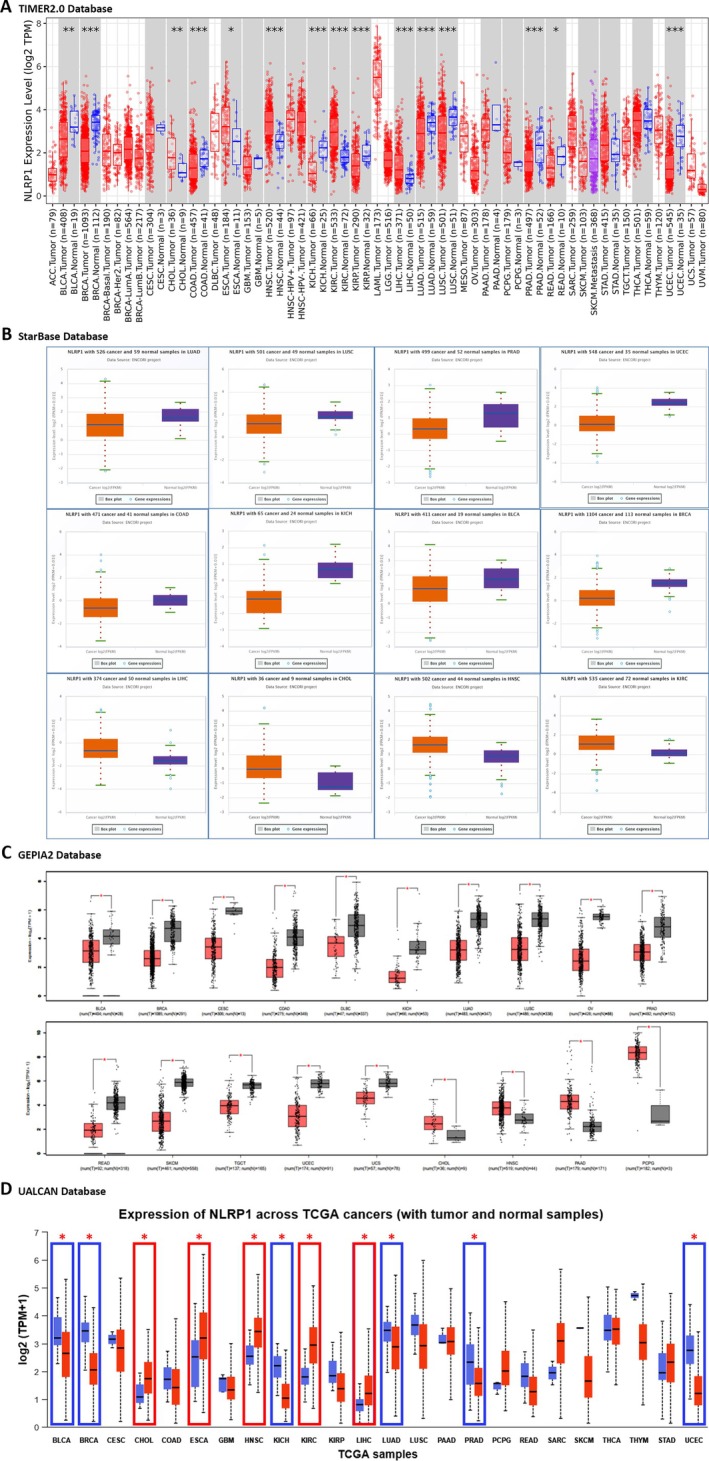
The expression pattern of NLRP1 in TCGA tumor types from various databases. (A) The expression status of NLRP1 in various tumor types was visualized through TIMER2.0 (*p < 0.05; **p < 0.01; ***p < 0.001). (B) Up and downregulated transcriptional level of NLRP1 based on the starBase database. The orange and blue boxes represent cancer and normal tissues, respectively (FDR < 0.05). (C) The expression pattern of NLRP1 based on GEPIA2 was shown as a boxplot in tumor tissues compared with match TCGA normal and GTEx data (*p < 0.05). (D) The Transcriptional pattern of NLRP1 at a pan‐cancer level from the UALCAN database (*p < 0.05).

For the differential expression analysis of NLRP1 in various cancer types compared to normal tissues, we used the TIMER2.0, starBase, GEPIA2, and UALCAN databases. According to TIMER2.0, NLRP1 mRNA expression exhibited a notable decrease in cancerous tissues across multiple cancer types when compared to their respective healthy counterparts. Specifically, tumor tissues of Breast Invasive Carcinoma (BRCA), Colon Adenocarcinoma (COAD), Kidney Chromophobe (KICH), Kidney Renal Papillary Cell Carcinoma (KIRP), Lung Adenocarcinoma (LUAD), Lung Squamous Cell Carcinoma (LUSC), Prostate Adenocarcinoma (PRAD), Uterine Corpus Endometrial Carcinoma (UCEC) (*p*‐value < 0.001), and Bladder Urothelial Carcinoma (BLCA) (*p*‐value < 0.01), as well as Rectum Adenocarcinoma (READ) (*p*‐value < 0.05), displayed a significant downregulation in NLRP1 expression. Conversely, elevated expression of NLRP1 was observed in Head and Neck Squamous Cell Carcinoma (HNSC), Kidney Renal Clear Cell Carcinoma (KIRC), Liver Hepatocellular Carcinoma (LIHC) (*p*‐value < 0.001), as well as Cholangio Carcinoma (CHOL) (*p*‐value < 0.01) and Esophageal carcinoma (ESCA) (*p*‐value < 0.05) when compared to normal tissues (Figure 2A, Table 1). Findings from the starBase database further validated the downregulated expression of NLRP1 in tumor tissues of BLCA, BRCA, COAD, KICH, LUAD, LUSC, PRAD, and UCEC, while KIRC, LIHC, CHOL, and HNSC exhibited higher expression levels relative to normal tissues, with FDR < 0.05 (Figure 2B, Table 1). The GEPIA2 database analysis revealed downregulation of the NLRP1 gene in tumor tissues of BLCA, BRCA, Cervical Squamous Cell Carcinoma and Endocervical Adenocarcinoma (CESC), COAD, Lymphoid Neoplasm Diffuse Large B‐cell Lymphoma (DLBC), KICH, LUAD, LUSC, Ovarian Serous Cystadenocarcinoma (OV), PRAD, READ, Skin Cutaneous Melanoma (SKCM), Testicular Germ Cell Tumors (TGCT), UCEC, and Uterine Carcinosarcoma (UCS). Conversely, it exhibited upregulation in CHOL, HNSC, Pancreatic Adenocarcinoma (PAAD), and Pheochromocytoma and Paraganglioma (PCPG) (*p*‐value < 0.05, and |log_2_fold change| > 1) (Figure 2C, Table 1). Additionally, insights derived from the UALCAN database supported the downregulation of NLRP1 in BLCA, BRCA, KICH, LUAD, LUSC, PRAD, and UCEC, while upregulation was observed in CHOL, ESCA, HNSC, KIRC, and LIHC (*p*‐value < 0.05) (Figure 2D, Table 1).

**TABLE 1:** The expression status of NLRP1 in various tumor types from different sources.

Database	NLRP1 expression pattern	
	Upregulation	Downregulation
TIMER2.0	CHOL‐ESCA‐HNSC‐KIRC‐LIHC	BLCA‐BRCA‐COAD‐KICH‐KIRP‐LUAD‐LUSC‐PRAD‐READ‐UCEC
starBase	CHOL‐HNSC‐KIRC‐LIHC	BLCA‐BRCA‐COAD‐KICH‐LUAD‐LUSC‐PRAD‐UCEC
GEPIA2	CHOL‐HNSC‐PAAD‐PCPG	BLCA‐BRCA‐CESC‐COAD‐DLBC‐KICH‐LUAD‐LUSC‐OV‐PRAD‐READ‐SKCM‐TGCT‐UCEC‐UCS
UALCAN	CHOL‐ESCA‐HNSC‐KIRC‐LIHC	BLCA‐BRCA‐KICH‐LUAD‐LUSC‐PRAD‐UCEC
Common	CHOL‐HNSC	BLCA‐BRCA‐KICH‐LUAD‐LUSC‐PRAD‐UCEC

**FIGURE 3:**
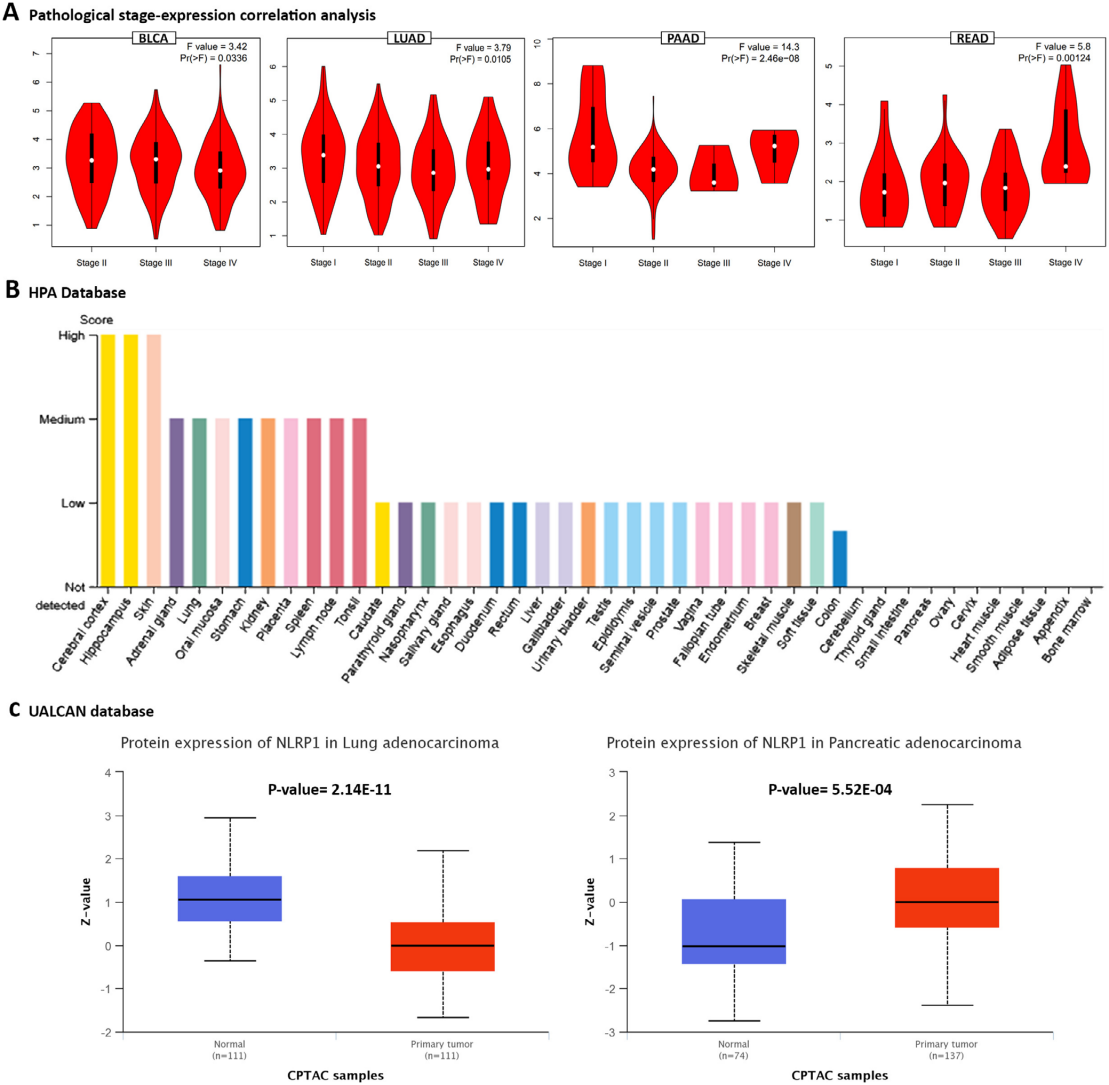
(A) NLRP1 expression in different stages of cancers BLCA, LUAD, PAAD, and READ. (B) Pan‐cancer NLRP1 protein expression in tumor tissues based on HPA. (C) Boxplot of NLRP1 protein expression in tumor tissues compared with normal tissues according to UALCAN.

The common tumor types with significantly downregulated or upregulated NLRP1 expression patterns were identified between TIMER2.0, starBase, GEPIA2, and UALCAN databases (Table 1). Our findings indicate a consistent downregulation of the NLRP1 gene in tumor tissues of BLCA, BRCA, KICH, LUAD, LUSC, PRAD, and UCEC, while notable overexpression of NLRP1 was observed specifically in CHOL and HNSC. Furthermore, our statistical analysis examining the significance of the differences observed between NLRP1 transcript levels and the pathological stage of various cancers revealed significant relationships solely in BLCA, LUAD, PAAD, and READ tumor tissues (*p*‐value < 0.05, Figure 3A).

### 3.3 Protein Expression Analysis

We utilized the HPA database to investigate the protein expression profile of NLRP1 in various normal tissues. Our findings revealed a notably high protein expression of NLRP1 in the cerebral cortex, hippocampus, and skin, as depicted in Figure 3B. Additionally, through the UALCAN database, we examined the protein levels of NLRP1 in various primary tumors compared to normal solid tissues. Our results indicated a significant elevation in NLRP1 protein expression in normal samples relative to primary tumors in lung adenocarcinoma patients. Conversely, in the case of pancreatic adenocarcinoma, we observed a substantial increase in NLRP1 protein level within primary tumors when compared to normal samples (Figure 3C). Understanding the implications of these expression levels is crucial, as they may influence key signaling pathways involved in cancer progression.

### 3.4 Survival Prognosis Analysis

**FIGURE 4:**
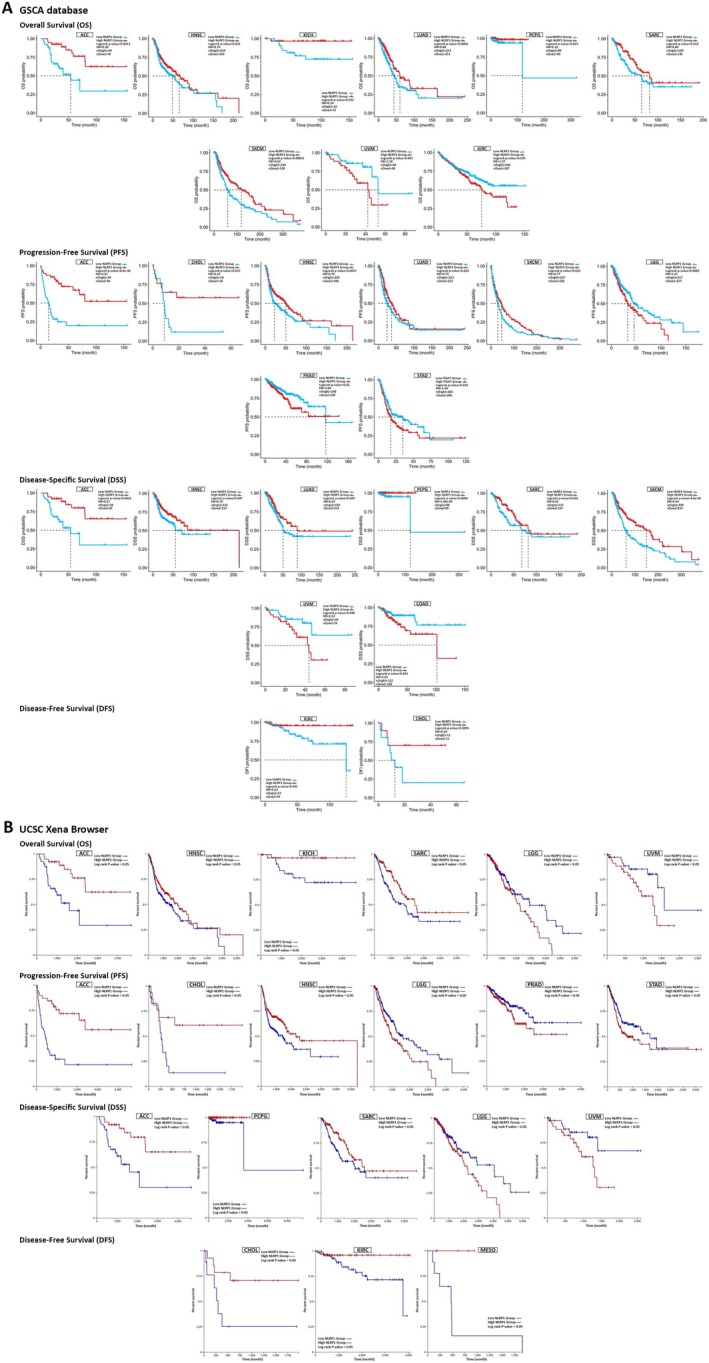
NLRP1 survival prognosis analysis. Various prognosis analyses including OS, PFS, DSS, and DFS were conducted using the GSCA database (A) and UCSC Xena Browser (B).

According to the dysregulation of NLRP1 in various malignancies, we postulate that its expression may be associated with the survival of patients with cancer. Our analysis utilized GSCA, GEPIA2, starBase, and UCSC Xena browser to examine the relationship between NLRP1 expression and diverse survival metrics, such as OS, DFS, PFS, and DSS. Through GSCA, a significant correlation was observed between NLRP1 downregulation and decreased OS in Adrenocortical Carcinoma (ACC) (*p* = 0.0013, HR = 0.28), HNSC (*p* = 0.031, HR = 0.74), KICH (*p* = 0.032, HR = 0.14), LUAD (*p* = 0.0066, HR = 0.66), PCPG (*p* = 0.024, HR = 0.12), Sarcoma (SARC) (*p* = 0.012, HR = 0.60), and SKCM (*p* = 0.00041, HR = 0.62). Conversely, NLRP1 upregulation was associated with poor OS in Uveal Melanoma (UVM) (*p* = 0.041, HR = 2.38) and KIRC (*p* = 0.035, HR = 1.37). Moreover, there was a correlation between NLRP1 downregulation and adverse PFS in ACC (*p* = 8.8e‐06, HR = 0.24), CHOL (*p* = 0.017, HR = 0.33), HNSC (*p* = 0.0047, HR = 0.70), LUAD (*p* = 0.023, HR = 0.75), and SKCM (*p* = 0.023, HR = 0.77), while its upregulation was linked to worse PFS in Brain Lower Grade Glioma (LGG) (*p* = 0.0083, HR = 1.43), PRAD (*p* = 0.02, HR = 1.60), and Stomach Adenocarcinoma (STAD) (*p* = 0.018, HR = 1.40). DSS analysis revealed a connection between poor DSS and NLRP1 downregulation in ACC (*p* = 0.0016, HR = 0.27), HNSC (*p* = 0.047, HR = 0.70), LUAD (*p* = 0.029, HR = 0.65), PCPG (*p* = 0.0098, HR = 1.49e‐09), SARC (*p* = 0.032, HR = 0.62), and SKCM (*p* = 4.6e‐05, HR = 0.54). Conversely, the upregulation of NLRP1 was correlated with poor DSS in UVM (*p* = 0.048, HR = 2.43) and COAD (*p* = 0.041, HR = 2.01). Furthermore, DFS analysis demonstrated that NLRP1 downregulation had a meaningful relationship with a poor prognosis in KIRC (*p* = 0.041, HR = 0.23) and CHOL (*p* = 0.0095, HR = 0.18) (Figure 4A, Table 2).

**TABLE 2:** The relationship between NLRP1 dysregulation and poor prognosis in various TCGA databases.

	Upregulation with poor prognosis	Downregulation with poor prognosis
Overall survival (OS)		
GSCA	KIRC‐UVM	HNSC‐ACC‐LUAD‐SKCM‐SARC‐PCPG‐KICH
UCSC Xena Browser	LGG‐UVM	ACC‐HNSC‐KICH‐SARC
GEPIA2	—	HNSC‐LUAD‐PAAD‐SKCM
starBase	DLBC‐KIRC	HNSC‐ACC‐LUAD‐PAAD‐SKCM
Common	—	HNSC
Progression‐free survival (PFS)		
GSCA	LGG‐PRAD‐STAD	ACC‐CHOL‐HNSC‐LUAD‐SKCM
UCSC Xena Browser	LGG‐PRAD‐STAD	ACC‐CHOL‐HNSC
Common	LGG‐PRAD‐STAD	ACC‐CHOL‐HNSC
Disease‐specific survival (DSS)		
GSCA	COAD‐UVM	SKCM‐PCPG‐ACC‐LUAD‐SARC‐HNSC
UCSC Xena Browser	LGG‐UVM	ACC‐PCPG‐SARC
Common	UVM	ACC‐PCPG‐SARC
Disease‐free survival (DFS)		
GEPIA2	STAD	CHOL‐LUAD‐PAAD
GSCA	—	CHOL‐KIRC
UCSC Xena Browser	—	CHOL‐KIRC‐MESO
Common	—	CHOL

Utilizing the UCSC Xena Browser, we found that the downregulation of NLRP1 was correlated with diminished OS in ACC, HNSC, KICH, and SARC. Additionally, an elevated expression of NLRP1 was linked to poor OS in LGG and UVM (*p*‐value < 0.05). Furthermore, a notable correlation was established between NLRP1 downregulation and adverse PFS in ACC, CHOL, and HNSC, while NLRP1 upregulation was associated with detrimental PFS in LGG, PRAD, and STAD (*p*‐value < 0.05). Moreover, the expression of NLRP1 exhibited correlations with DSS in various human cancers. Indirectly, upregulated NLRP1 was associated with worse survival in LGG and UVM, while in ACC, PCPG, and SARC, its downregulation was directly linked to an adverse prognosis (*p*‐value < 0.05). Additionally, The DFS analysis indicated that reduced NLRP1 expression is associated with poorer DFS in CHOL, KIRC, and Mesothelioma (MESO). (*p*‐value < 0.05, Figure 4B, Table 2).

**FIGURE 5:**
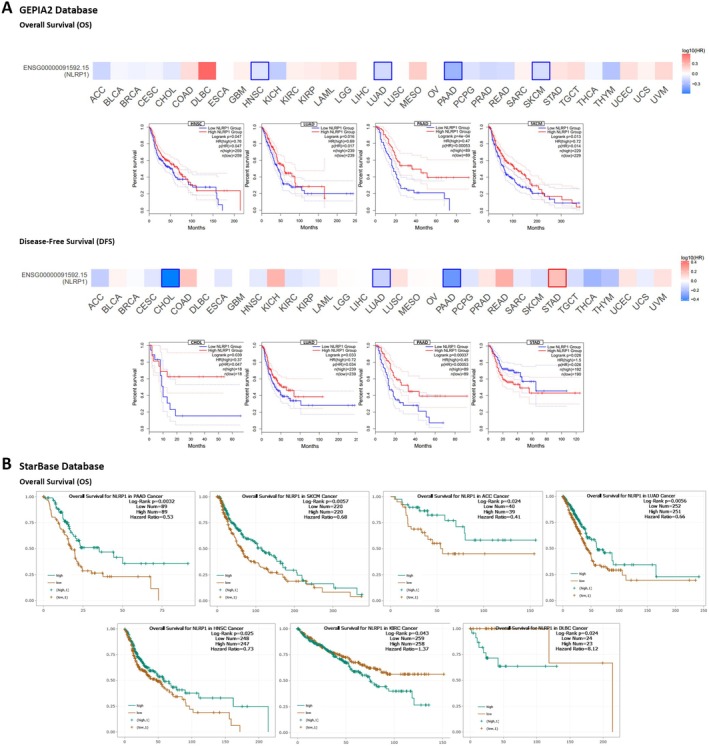
The relationship between NLRP1 gene expression and survival prognoses. (A) GEPIA was used to conduct OS and DFS analysis as well as their survival maps. (B) OS prognosis analysis in various tumor types using the starBase database.

In the GEPIA2 database, we stratified the samples into NLRP1 high‐expression and low‐expression categories based on the NLRP1 median transcription level, subsequently evaluating the association between NLRP1 expression and both OS and DFS. The findings revealed that low NLRP1 expression was associated with poor OS in HNSC (*p* = 0.047, HR = 0.76), LUAD (*p* = 0.016, HR = 0.69), PAAD (*p* = 4e‐04, HR = 0.47), and SKCM (*p* = 0.013, HR = 0.72). Additionally, the analysis of DFS data demonstrated that decreased NLRP1 transcription levels correlated with poor prognosis in various cancers, including CHOL (*p* = 0.039, HR = 0.37), LUAD (*p* = 0.033, HR = 0.72), and PAAD (*p* = 0.00037, HR = 0.45). Furthermore, NLRP1 upregulation was linked to adverse DFS in STAD (*p* = 0.026, HR = 1.5) (Figure 5A, Table 2). Alternatively, the starBase database highlighted the association between NLRP1 downregulation and diminished OS in PAAD (*p* = 0.0032, HR = 0.53), SKCM (*p* = 0.0057, HR = 0.68), ACC (*p* = 0.024, HR = 0.41), LUAD (*p* = 0.0056, HR = 0.66), and HNSC (*p* = 0.025, HR = 0.73), while NLRP1 upregulation was related to adverse OS in KIRC (*p* = 0.043, HR = 1.37) and DLBC (*p* = 0.024, HR = 8.12) (Figure 5B, Table 2). Ultimately, an intersection analysis of all survival prognosis data groups revealed that NLRP1 downregulation was correlated with poorer OS in HNSC. Additionally, the decreased NLRP1 expression level was linked to adverse PFS in ACC, CHOL, and HNSC, while increased NLRP1 expression was associated with detrimental PFS in LGG, PRAD, and STAD. Furthermore, ACC, PCPG, and SARC displayed worse DSS prognosis in correlation with NLRP1 downregulation, whereas UVM exhibited poor DSS in correlation with NLRP1 overexpression. CHOL emerged as the only cancer type displaying an association between NLRP1 downregulation and poor DFS (Table 2).

### 3.5 Drug Sensitivity Analysis

**FIGURE 6:**
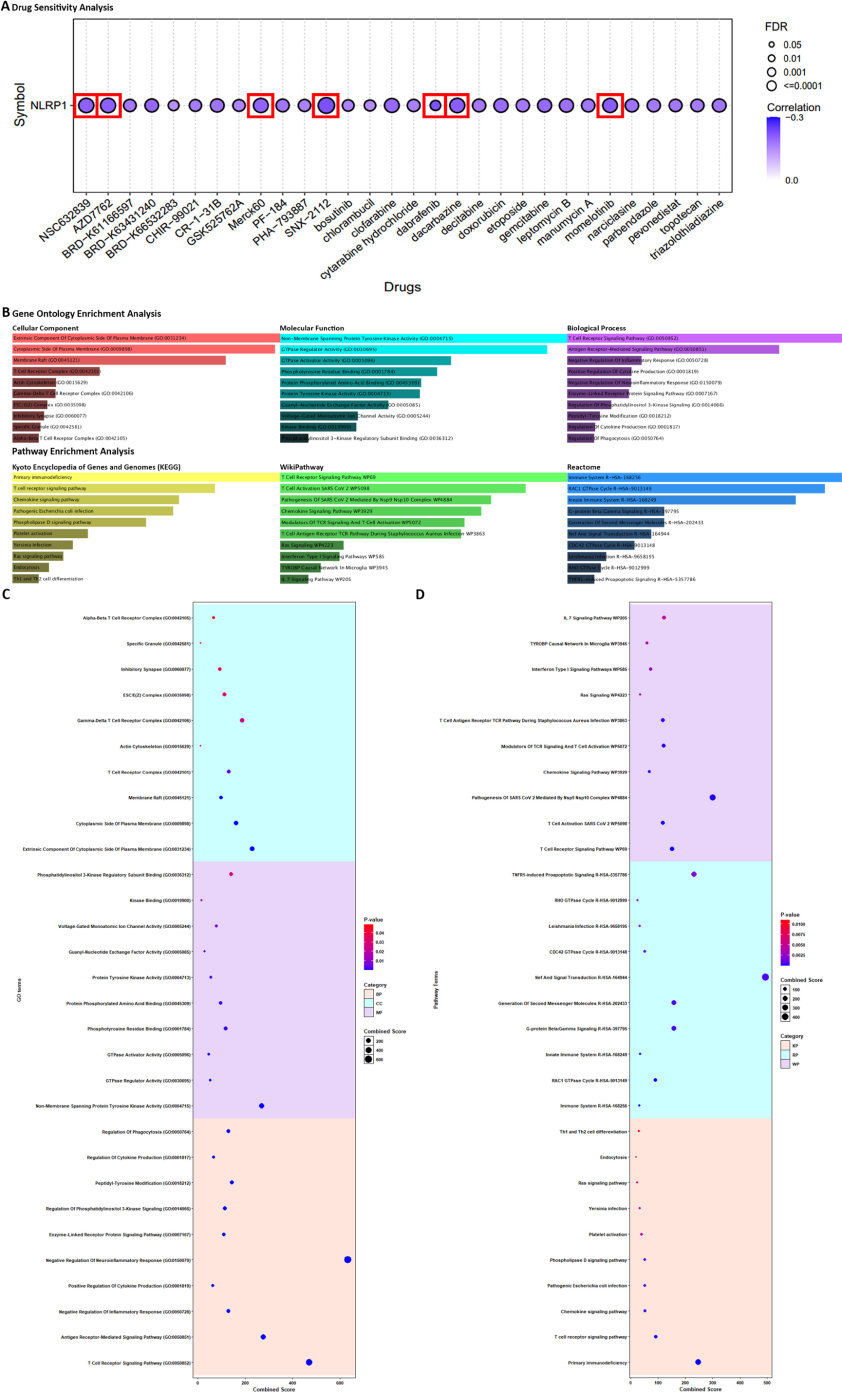
The NLRP1‐related drug sensitivity and gene set enrichment analysis. (A) The drug sensitivity analysis for the NLRP1 gene using data from the GSCA database shows the correlation between NLRP1 gene expression and drug sensitivity. (B) The bar plots and (C, D) dot plots of GO and pathway enrichment analysis from the Enrichr tool.

Drug sensitivity analysis revealed that cancer patients with low‐expression levels of the NLRP1 gene demonstrated heightened sensitivity to SNX‐2112, Dabrafenib, Momelotinib, Dacarbazine, AZD7762, NSC632839, and Merck60 (*r* value < −0.2, FDR < 0.001) based on the findings from the CTRP dataset (Figure 6A). SNX‐2112 is a highly selective inhibitor of Hsp90 that effectively suppresses tumor cell proliferation, angiogenesis, and osteoclast formation in multiple myeloma and other hematologic malignancies [34]. Dabrafenib is a kinase inhibitor used for treating patients with unresectable or metastatic melanoma, metastatic non‐small cell lung cancer, locally advanced or metastatic anaplastic thyroid cancer, pediatric low‐grade glioma, and other solid tumors harboring specific BRAF mutations [35]. Momelotinib is a small‐molecule inhibitor of Janus kinase (JAK) designed for the treatment of primary or secondary myelofibrosis in patients at intermediate or high risk [36]. Dacarbazine is a non‐specific cell cycle alkylating agent utilized in the management of metastatic malignant melanoma [37]. NSC632839 is an inhibitor of Ubiquitin C‐Terminal Hydrolase L1 that significantly induces protein light chain 3 puncta formation and modulates autophagy [38]. Merck60 is a highly potent and selective inhibitor of HDAC1 and HDAC2 that influences histone deacetylation [39]. For a comprehensive overview of these correlations between NLRP1 expression and drug sensitivity, refer to Figure 6A, which visually represents the complex relationship between NLRP1 and drug responses (*r* value < −0.15, FDR < 0.05).

### 3.6 Functional Enrichment Analysis

The results of our analysis elucidate a pronounced participation of the delineated genes in an array of critical cellular mechanisms. These genes are discovered to exert pivotal functions within a range of signaling pathways, such as the T Cell Receptor Signaling Pathway, the receptor‐mediated Signaling Pathway, and pathways governing the Negative Regulation of Inflammatory Response and the Regulation of Cytokine Production. They are also integral in modulating pathways like the Regulation of Phosphatidylinositol 3‐Kinase Signaling and thus underscore their instrumental roles in modulating inflammatory signaling cascades. In addition, our examination has exposed that a significant fraction of these genes is associated with a spectrum of molecular activities, notably including Non‐Membrane Spanning Protein Tyrosine Kinase Activity, GTPase Regulator Activity, and Kinase Binding. In terms of cellular localization, the genes are predominantly localized to the Cytoplasmic Side of the Plasma Membrane and intrinsically within the T Cell Receptor Complex, suggesting their active participation in signal transduction pertinent to immune responses. Furthermore, our analysis delves beyond the scope of functional annotations to incorporate a comprehensive examination of the involvement of the identified genes in distinct biological pathways. Notably, we have determined significant pathways by leveraging comprehensive databases, such as KEGG, Wikipathway, and Reactome. These pathways include the T cell receptor signaling pathway, the chemokine signaling pathway, and other pathways that play essential roles within the broader framework of the immune system. These pathway associations provide valuable insights into the potential implications of the identified genes in shaping immune system functionality (Figure 6).

### 3.7 Cancer‐Associated Fibroblast Infiltration Analysis

**FIGURE 7:**
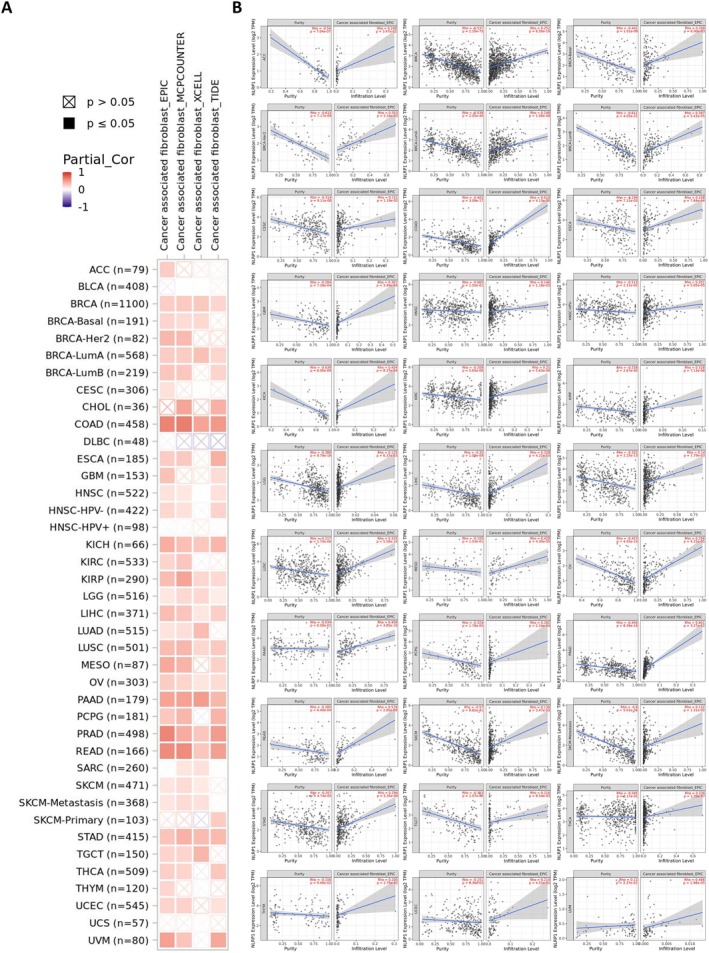
CAF infiltration analysis. (A) The correlation chart between NLRP1 gene expression and CAF infiltration with 4 different algorithms among TCGA cancer types without purity adjustment. (B) The purity‐adjusted EPIC results of all TCGA cancers in which a significant correlation has been observed.

Previous studies have demonstrated the involvement of CAF in adjusting the behavior of diverse neoplasm‐infiltrating immune cells [40]. The infiltration of CAFs may further modulate the role of NLRP1 in the tumor microenvironment. To investigate the association between CAF infiltration and NLRP1 expression, we employed the TIMER2.0 database. Figure 7A displays the outcomes before purity adjustment, revealing a positive correlation between NLRP1 expression and diverse TCGA cancers. Tumor purity emerges as a significant confounding element in this investigation, as the majority of immune cell types exhibit negative correlations with tumor purity. Consequently, upon implementing the purity‐adjusted EPIC algorithm, the analysis revealed a negative correlation between NLRP1 expression and CAFs in ACC, BRCA, CESC, COAD, ESCA, Glioblastoma Multiforme (GBM), HNSC, KICH, KIRC, KIRP, LGG, LIHC, LUAD, LUSC, MESO, OV, PAAD, PCPG, PRAD, READ, SKCM, STAD, TGCT, Thyroid Carcinoma (THCA), Thymoma (THYM), and UCEC. Additionally, a negative association was identified between NLRP1 transcriptional level and CAF in specific subgroups, such as HPV‐negative HNSC, BRCA basal cell carcinoma, BRCA HER2 positive, BRCA‐lumA, BRCA‐lumB, and SKCM metastasis based on the EPIC algorithm. Conversely, a positive correlation has been observed in UVM (Figure 7B).

### 3.8 Genetic Alteration Analysis

**FIGURE 8:**
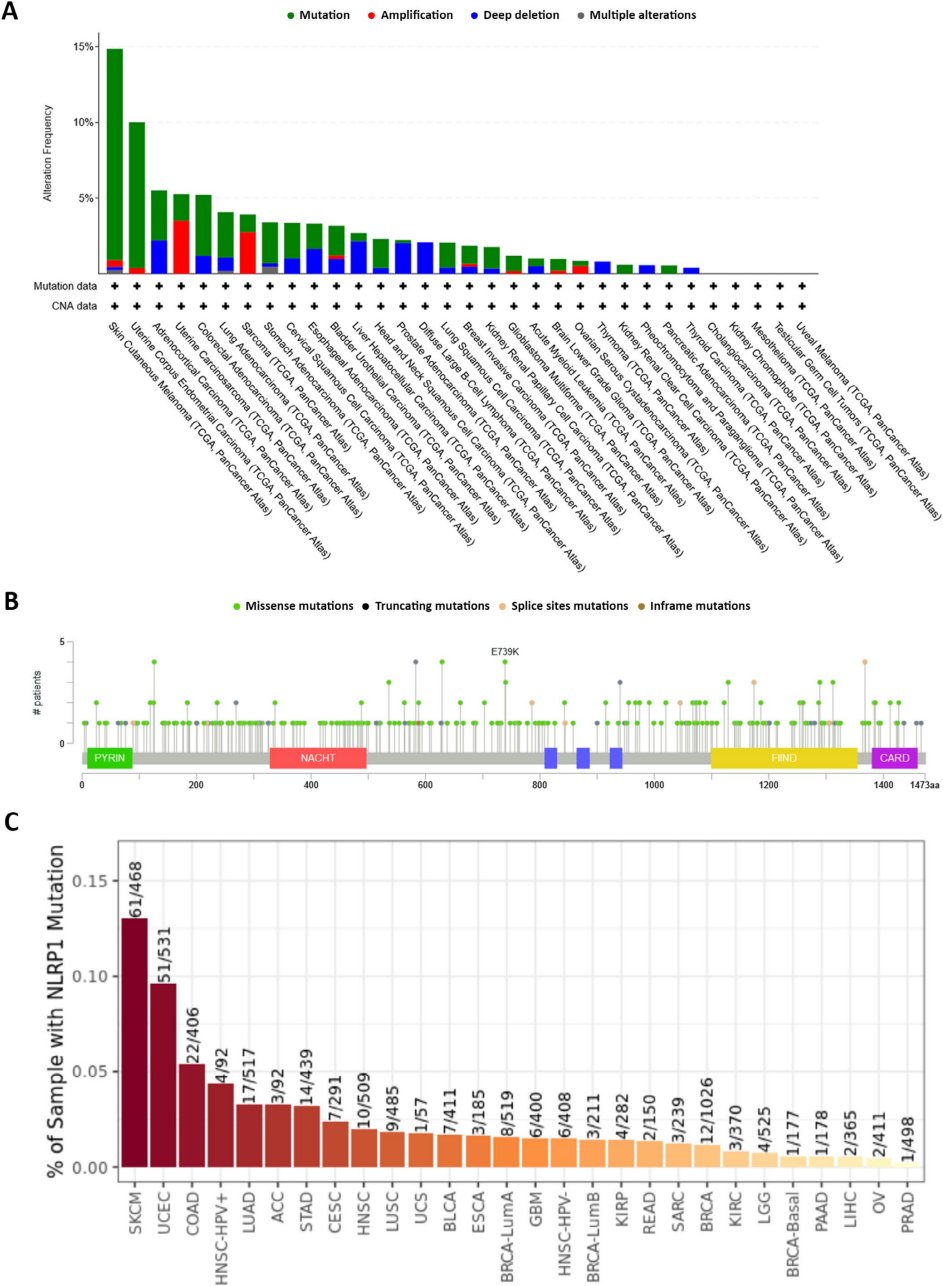
NLRP1 genetic alteration analysis. (A, B) The various alteration and mutation types in different malignancies. (C) The mutation distribution in patients with diverse cancers.

Using cBioPortal, we examined the genetic alterations of NLRP1 across 32 TCGA tumor types. Notably, the highest genetic alteration frequencies in the NLRP1 gene were observed in SKCM, UCEC, and ACC, with alteration frequencies of 14.86%, 10.02%, and 5.49%, respectively. Moreover, SKCM, ACC, and UCS tumor subjects exhibited the highest frequencies of NLRP1 mutations (13.96%), deep deletions (2.2%), and amplifications (3.51%), respectively. Several alterations were detected with frequencies < 1% (Figure 8A, Data S3). A total of 303 mutations were identified in the NLRP1 gene across TCGA tumor subjects, comprising 243 missenses, 44 truncating mutations, 15 splice site alterations, and 1 in‐frame mutation. These mutations were distributed throughout the NLRP1 gene, encompassing both domain and non‐domain sites (Figure 8B, Data S4). Additionally, we utilized TIMER2.0 to further scrutinize NLRP1 gene mutations across various human malignancies. The NLRP1 mutations were predominantly observed in patients with SKCM (61 out of 468 patients), UCEC (51 out of 531 patients), and COAD (22 out of 406 patients) (Figure 8C).

### 3.9 Protein–Protein Interaction Analysis

**FIGURE 9:**
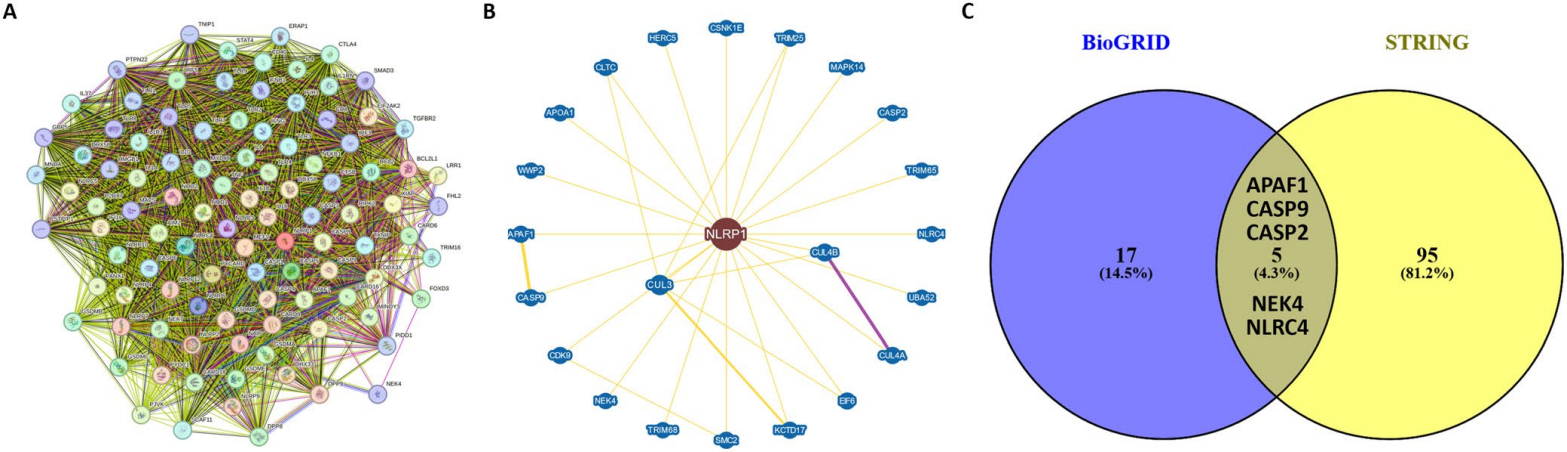
NLRP1 PPI analysis. (A) The PPI network of 100 genes correlates physically and functionally with NLRP1 by the STRING tool. (B) NLRP1‐protein interactions obtained by BioGRID. The violet line represents both physical and genetic interactions, while the yellow line indicates only physical interactions. (C) Intersection analysis of NLRP1‐related gene displaying by Venny 2.1.

The STRING database was employed to extract the top 100 genes exhibiting both functional and physical interaction with NLRP1, as illustrated in Figure 9A. Subsequently, by employing the BioGRID database, we identified 22 genes that were physically associated with NLRP1. This set comprised 14 genes (WWP2, CDK9, APOA1, TRIM68, EIF6, CUL3, UBA52, CLTC, SMC2, CSNK1E, CUL4B, NEK4, KCTD17, CUL4A) curated from high‐throughput (HTP) studies, and seven genes (CASP2, HERC5, NLRC4, TRIM65, CASP9, APAF1, MAPK14) obtained from low throughput (LTP) research, with only one gene (TRIM25) curated from both HTP and LTP sources (Figure 9B). The intersection of the genes from STRING and BioGRID was determined and visualized using Venny 2.1 (Figure 9C), leading to the identification of five genes (APAF1, CASP2, CASP9, NEK4, and NLRC4) with literature‐confirmed interactions with NLRP1.

### 3.10 Methylation

When utilizing the DNMIVD database to explore the methylation pattern of the NLRP1 gene's promoter across 22 distinct cancer types, we determined significant hypomethylation in the promoter region of the NLRP1 gene in ESCA (Beta difference = −0.219818 and adjusted *p*‐value = 0.000117) and PAAD (Beta difference = −0.202757 and adjusted *p*‐value = 0.003035) (Data S5). Moreover, the results of the correlation study revealed a notable inverse association between the level of methylation in the promoter region and the expression level of the NLRP1 gene in ESCA (Pearson *p*‐value = 4.0513e‐06, Pearson *R* value = −0.344137, Spearman *p*‐value = 6.55e‐8, Spearman *R* value = −0.398828) (Data S6).

## 4 Discussion

NLRP1 serves as a sensor for intracellular danger signals, prompting the assembly of multiprotein complexes called inflammasomes. This cascade of events ultimately activates caspase‐1‐dependent pyroptosis [41]. Activation of the inflammasome represents a crucial signaling pathway contributing to both acute and chronic inflammation, exerting a significant influence on all stages of tumorigenesis [42]. Acknowledging the pivotal role of NLRP1 in cancer pathogenesis and recognizing the uncertainties surrounding its alterations in various cancers, we conducted a comprehensive investigation utilizing diverse databases to explore different aspects of NLRP1 across various cancer types in the TCGA project. Our analysis of gene expression unveiled a decrease in NLRP1 levels in BLCA, BRCA, KICH, LUAD, LUSC, PRAD, and UCEC tumor tissues compared to normal ones. Conversely, NLRP1 was found to be overexpressed, particularly in CHOL and HNSC cancer types. Additionally, our study revealed statistically significant associations between the transcriptional level of NLRP1 and the pathological stage of BLCA, LUAD, PAAD, and READ tumor tissues. Wei et al. demonstrated that the transcriptional level of NLRP1 was elevated in primary breast neoplasm tissue compared to nearby noncancerous tissue [10]. Furthermore, through quantitative real‐time polymerase chain reaction (qRT‐PCR), elevated expression of NLRP1 was demonstrated in HNSC tissue compared to adjacent normal tissue in patients [43]. Furthermore, a multi‐omics analysis by Chen et al. revealed that NLRP1 levels were increased in CHOL and LIHC compared to normal tissues [44]. Several additional multi‐omics analyses exploring pyroptosis‐related genes in lung adenocarcinoma consistently showed downregulation of NLRP1 in LUAD tissues compared to normal tissues [45, 46, 47, 48]. Consistent with previous studies, our findings highlight the tumor‐suppressive influence of NLRP1 in the majority of TCGA cancer types.

In our analysis, NLRP1 protein expression decreased in LUAD, while it increased in PRAD when comparing primary tumors to normal samples. While there is no specific study focusing on NLRP1 protein expression in lung adenocarcinoma and prostate adenocarcinoma compared to normal tissues, Liang et al. reported a substantial increase in NLRP1 protein level in patients with prostate cancer compared to those with prostate hyperplasia. Notably, the protein expression levels of NLRP1 showed a positive correlation with the Gleason score of prostate cancer [49]. Although NLRP1 RNA expression was downregulated in both LUAD and PRAD, the corresponding decrease in protein levels observed in LUAD was expected; however, the increase in protein levels in PRAD was unexpected. This discrepancy could be explained by the effective post‐transcriptional regulation of NLRP1, which may lead to decreased RNA translation in normal tissues.

Our investigation of survival prognosis reveals that reduced NLRP1 expression is correlated with inferior OS in HNSC. Conversely, elevated levels of NLRP1 are linked to reduced PFS in LGG, PRAD, and STAD, while decreased expression of NLRP1 is associated with a poorer PFS prognosis in ACC, CHOL, and HNSC. Regarding DSS, heightened NLRP1 expression is indicative of an unfavorable prognosis in UVM, whereas a substantial decrease in NLRP1 expression leads to poor DSS in ACC, PCPG, and SARC. Furthermore, the downregulation of NLRP1 is correlated with unfavorable DFS in CHOL. Several studies have explored the potential of NLRP1 as a prognostic biomarker in various cancers, including lung adenocarcinoma [45, 46, 50], gastric cancer [51], prostate cancer [49], pancreatic cancer [52, 53], head and neck squamous cell carcinoma [54], and laryngeal carcinoma [55]. Shen et al. confirmed that decreased expression of NLRP1 was significantly associated with a poor prognosis of LUAD [47]. Consistent with prior research, our results demonstrate the potential of NLRP1 as a prognostic biomarker in various types of cancer.

Cancer cells develop resistance to drugs through a complex process involving crucial contributors like nuclear factor‐κB (NF‐κB) and IL‐1β. Zhai et al. explored the connection between the inflammasome sensor NLRP1 and acquired drug resistance to temozolomide in melanoma, as NACHT, LRR, and PYD domains‐containing protein inflammasomes play a role in IL‐1β maturation and NF‐κB activation. Their findings indicated heightened expression of NLRP1 in melanoma cells that had become resistant to temozolomide [56]. Conversely, diminished levels of NLRP1 increased the sensitivity of pancreatic cancer cells to ERK inhibitors [52]. Multi‐omics analyses further reinforce the prospect of NLRP1 serving as a biomarker for drug sensitivity in pancreatic adenocarcinoma [57, 58] and head and neck squamous cell carcinoma [54]. Our comprehensive pharmacogenomic assessment revealed a significant correlation between reduced expression of the NLRP1 gene in cancer patients and enhanced sensitivity to a spectrum of chemotherapeutic and targeted agents. Specifically, individuals with diminished NLRP1 expression levels were found to be more responsive to SNX‐2112, Dabrafenib, Momelotinib, Dacarbazine, AZD7762, NSC632839, and Merck60. These insights, in line with previous studies, underscore the potential of NLRP1 as a predictive biomarker for chemotherapy response, paving the way for personalized treatment regimens that leverage the heightened drug efficacy in these patient subsets.

The initiation of inflammasome activation constitutes a pivotal mechanism for the innate immune response, culminating in the release of bioactive IL‐1β and IL‐18 [59]. These potent proinflammatory cytokines are instrumental in instigating pyroptosis, an inflammatory programmed cell death [4]. Zhai et al. elucidate that the NLRP1 inflammasome plays a dual role in melanoma progression by promoting inflammasome activation, which in turn augments the pyroptotic cell death pathway while simultaneously impeding apoptotic mechanisms, thus contributing to tumor growth [60]. Furthermore, our functional enrichment analysis delineates an essential involvement of NLRP1 and its associated genes in regulating the T‐cell receptor‐signaling pathway along with the Chemokine signaling pathway, both of which are integral to regulating an effective immune response. Additionally, these entities participate in a myriad of other pathways that are imperative for the comprehensive and robust functionality of the immune defense system. Such interactions may have extensive implications, influencing various cellular processes ranging from immune surveillance to cell proliferation and death, potentially affecting disease pathology and therapeutic responses.

In the tumor microenvironment, CAFs have been demonstrated to fulfill various functions in tumor development. They release growth factors, inflammatory ligands, and extracellular matrix proteins as well as increase cancer cell proliferation, therapy resistance, and immune exclusion. Nevertheless, recent research suggests that in certain situations, CAFs may exert a restraining influence on tumor progression [61]. Thoroughly understanding the tumor‐promoting and tumor‐restraining roles of different CAF subtypes, including the evolution of these complex bimodal functions and how neoplastic cells control them during cancer progression, could aid in the development of innovative diagnostic and therapeutic strategies [62]. Our analysis of CAF infiltration unveiled a negative correlation between NLRP1 expression and the presence of CAFs in ACC, BRCA, CESC, COAD, ESCA, GBM, HNSC, KICH, KIRC, KIRP, LGG, LIHC, LUAD, LUSC, MESO, OV, PAAD, PCPG, PRAD, READ, SKCM, STAD, TGCT, THCA, THYM, and UCEC. This inverse relationship indicates that NLRP1 may play a potentially suppressive role in the recruitment or propagation of CAFs within the tumor microenvironment. Additionally, the variable expression of NLRP1 influences the heterogeneity and functional phenotype of CAFs, implicating it as a pivotal factor in the tumoral stroma and a conceivable prognostic biomarker or target for therapeutic intervention.

Through the systematic investigation of The TCGA datasets, we discerned a compendium of 303 genetic aberrations within the NLRP1 gene locus. These anomalies were predominantly missense, truncating, splice site, and in‐frame mutations, respectively. The distribution of these mutations spanned the entire genomic architecture of NLRP1, afflicting regions integral to functional domains as well as segments external to these specified areas. Notably, the most pronounced incidences of genetic alterations were found within cases with SKCM, UCEC, and ACC. In a case–control study conducted in Sweden, findings revealed a notable prevalence of the NLRP1 variant (rs12150220) among female melanoma patients with fair skin, demonstrating robust correlations with nodular melanoma [8]. Lee et al. identified a frameshift mutation (c.1748delA) in the NLRP1 gene, which was associated with elevated microsatellite instability in colon cancer [63]. These data reveal a complex landscape of NLRP1‐related genetic variability that may contribute to cancer development across different types.

Our PPI analysis unveiled five genes—APAF1, CASP2, CASP9, NEK4, and NLRC4—that exhibit interactions with NLRP1. It is important to note that the specific factor responsible for directly binding to and activating NLRP1 has yet to be identified. The inflammasomes share similarities with the apoptosome, featuring APAF1. APAF1 protein consists of an N‐terminal CARD, a central NBD, and C‐terminal WD‐40 repeats, reflecting the domain composition of the NLR protein family in some aspects [64]. NLRP1 was first characterized for its ability to modulate apoptotic pathways, accomplishing this either through direct binding to initiator caspases, such as CASP2 and CASP9, or indirectly, by engagement with APAF1, thereby potentiating the functional capabilities of the apoptosome [65]. This expansion of our understanding asserts the complexity of NLRP1's involvement, accentuating its multifactorial influence not only in the apoptotic process but, by extension, in the nuanced regulatory mechanisms governing cell survival and death. Therefore, the strategic manipulation of NLRP1's interactive network could offer avenues for innovative treatments, with potential applications in controlling aberrant cellular proliferation, such as that observed in tumorigenic conditions, or in modulating programmed cell death for therapeutic benefit.

Recently, there has been a suggestion that epigenetic regulation, particularly CpG DNA methylation, serves as a complementary mechanism for controlling inflammasome activity. The modulation of inflammasome expression through epigenetic mechanisms, involving either repression or promotion, plays a considerable function in developing diseases with varying degrees of severity [66]. Sand et al. demonstrated that the expression of NLRP1 is silenced through methylation in Squamous Cell Carcinoma cell lines SCC13 and A431 [11]. Through genomic analysis, we discerned substantial hypomethylation within the promoter locus of the NLRP1 gene, specifically in ESCA and PAAD. Subsequently, our correlational assessments revealed an inverse relationship between promoter methylation status and NLRP1 gene expression within ESCA. These findings open novel possibilities for therapeutic intervention, suggesting that modulation of DNA methylation patterns could serve as a strategic approach to managing aberrant NLRP1 expression and its associated proinflammatory responses in oncogenesis, enhancing personalized medicine strategies for affected patients.

## 5 Conclusion and Prospect

Our comprehensive analysis has revealed a consistent downregulation of NLRP1 across multiple cancer types, suggesting a potential role for NLRP1 as a tumor suppressor. This downregulation is generally associated with poor prognosis, highlighting the significance of NLRP1 in cancer progression and patient survival. Moreover, our findings have unveiled correlations between NLRP1 expression and various drug sensitivities. These associations suggest the potential utility of NLRP1 as a promising diagnostic, prognostic, and predictive biomarker in diverse cancers, providing valuable insights for personalized treatment strategies. Furthermore, we have identified potential molecular mechanisms through which NLRP1 may influence cancer‐associated fibroblast immune infiltration, immune cell functionality, and participation in cancer progression. However, integrating clinical data with experimental research will be crucial in establishing NLRP1 as a reliable diagnostic, prognostic, and predictive biomarker, as well as a promising therapeutic target.

## Author Contributions

**Leila Habibipour:** conceptualization (equal), formal analysis (equal), methodology (equal), writing – review and editing (equal). **Mahboubeh Sadeghi:** conceptualization (equal), methodology (equal), software (equal), writing – review and editing (equal). **Alireza Raghibi:** writing – review and editing (equal). **Nima Sanadgol:** writing – review and editing (equal). **Amirhossein Mohajeri Khorasani:** conceptualization (equal), data curation (equal), formal analysis (equal), methodology (equal), software (equal), validation (equal). **Pegah Mousavi:** project administration (equal), supervision (equal), writing – review and editing (equal).

## Ethics Statement

The authors have nothing to report.

## Consent

The authors have nothing to report.

## Conflicts of Interest

The authors declare no conflicts of interest.

## Supporting information


**Figure S1.** NLRP1 expression pattern in various normal tissues based on HPA, GTEx, and FANTOM5 datasets.

**Data S1.** Comprehensive details of TCGA cancer types and Genotype‐Tissue Expression (GTEx) normal tissues included in the study.

**Data S2.** The top 100 co‐expressed genes with *NLRP1*, identified using the ARCHS4 RNA‐seq gene–gene co‐expression matrix.

**Data S3.** Frequency, type, and count of *NLRP1* gene alterations across various cancers, based on TCGA PanCancer Atlas data.

**Data S4.** Complete information of *NLRP1* gene mutations identified in TCGA cancer types.

**Data S5.** Statistical summary of the methylation pattern analysis of the *NLRP1* gene promoter across different TCGA cancer types.

**Data S6.** The correlation between *NLRP1* promoter methylation level and its gene expression across various TCGA cancer types.

## Data Availability

The datasets used and/or analyzed during the current study are available from the corresponding author upon reasonable request.
